# System Dynamics Analysis of Upper Echelons’ Psychological Capital Structures in Chinese Mixed-Ownership Reform Enterprises During the COVID-19 Pandemic

**DOI:** 10.3389/fpsyg.2022.948203

**Published:** 2022-06-28

**Authors:** Yilei Jiao, Yuhui Ge, Huijuan Liu

**Affiliations:** ^1^College of Management, University of Shanghai for Science and Technology, Shanghai, China; ^2^College of Management, Jiaxing Nanhu University, Jiaxing, China; ^3^Business College, Shanghai University of Finance and Economics, Shanghai, China

**Keywords:** system dynamics, Chinese mixed-ownership reform enterprise, upper echelon, psychological capital structure, behavioral conflict

## Abstract

The COVID-19 pandemic has caused major changes in the psychological capital structure of individuals and groups, especially among members of the upper echelons of Chinese mixed-ownership reform enterprises, who are more sensitive to the environment. Based on prospect theory. In order to further study the changes in the psychological capital structure of upper echelons of the mixed ownership reform of state-owned enterprises under the influence of the COVID-19, and what impact it has on the decision-making behavior of the upper echelons and the development performance of the mixed ownership reform enterprises, this paper introduces the system dynamics research method into the research field of the upper echelons for the first time, and studies the psychological capital structure of the upper echelons through simulation. This paper puts forward new research ideas for the research on the psychological capital structure of upper echelons. Using the system dynamics method, this study investigates the changes induced by the COVID-19 pandemic, on the psychological capital structure of the upper echelons of Chinese mixed-ownership reform enterprises; and assesses the concept model of behavioral psychological capital adjustment. The impact of COVID-19 on communication strategies among upper echelons, directly affects the evolution results of the decision-making system. The psychological capital structure in the upper echelons has evolutionary stability strategies in three cases. In some cases, the system evolution presents periodic characteristics. The higher the probability of group communication, the more stable the psychological capital structure, and the greater the fluctuation of behavioral integration. There is a significant correlation between the level of efficacy and resilience of upper echelons psychological capital structure and upper echelons decision-making behavior. Under the condition of improving the communication probability among upper echelon members, there is a positive correlation between the level of hope and optimism of upper echelon and the power structure of upper echelon and the development performance of mixed reform enterprises. Develop the psychological capital structure of upper echelon of mixed reform enterprises, improve the level of financing development decision-making ability and improve decision-making performance.

## Introduction

On May 18, 2020, the opinions of the Communist Party of China (CPC) Central Committee and the State Council stated that the reform of mixed ownership of state-owned enterprises should be actively and steadily promoted to accelerate the improvement of the socialist market economic system in the new era. This is considered conducive to learning from each other’s strengths, mutual promotion, and the common development and expansion of the function of the capital, while simultaneously maintaining and increasing value, improving competitiveness, and enhancing the vitality, control, and influence of the national economy.

The research and practice results of mixed reform on corporate governance, mixed reform enterprise behavior, and performance management point out that the high-quality development of enterprises is inseparable from scientific and reasonable power structure arrangement. The traditional source of power structure change of mixed-ownership enterprises mainly comes from the change in equity proportion, but its limitations are also obvious. The change in equity proportion leads to the rapid convergence of the performance improvement of mixed reform, however the performance improvement efficiency of mixed reform has not been fully realized. The next important breakthrough of mixed-ownership reform will be to continuously improve performance and provide a scientific upper echelon power structure design for mixed-ownership enterprises.

On March 11, 2020, Tam Desai, Director-General of the World Health Organization declared COVID-19 as a pandemic. The pandemic has had a strong negative impact on the global economy and society, causing financial markets to fluctuate. There are signs of a recession owing to the volatility of global economy in the face of an economic depression, and COVID-19 could cost the world economy trillions of dollars in the next 5 years. The pandemic has directly impacted the real economy, especially tourism, air transport, retail, and other service and manufacturing industries, triggering financial market turmoil, and shutting down many small- and medium-sized enterprises. This has caused severe price shocks in some products, greatly impacting the global economy, including the world’s major economies (Ahmed et al., 2020). In addition to the economic impacts, COVID-19 has psychologically impacted individuals worldwide. Restubog et al. (2020) discuss the implications of COVID-19 for maintaining individual psychological well-being and employment security while also managing family and work responsibilities. The literature on emotion regulation provides evidence to help mitigate the downstream negative consequences of COVID-19 on people’s work lives. According to Wu et al. (2020), and Wang et al. (2020) the emotional, cognitive, physical, and mental response of staff reveals an obvious “exposure effect,” which calls for a psychological crisis intervention strategy. According to Jiao et al. (2021), the game of group decision-making in the top management team provides offers new ideas for breakthroughs in this area to study the evolution process of internal conflict in the top management team using game theory, model the process of internal behavior conflict resolution in the top management team using the cybernetics method, and analyze the game and control of group decision-making in the top management team using the system dynamics method.

The development of mixed-ownership reform of state-owned enterprises in China has experienced four stages: germination, birth, growth and maturity. Budding period: the time span is from the late 1970s to the early 1990s. Led by the advanced technology and abundant funds of state-owned enterprises, non-public economies such as the private economy quietly sprouted. Birth period: the time span is from the early 1990s to the middle and late 1990s. It takes the foreign economy as the leading factor, combines with various economic forms, and adopts more advanced technology, equipment and management concepts to drive and improve the technological progress and management level of relevant enterprises. Growth period: it began in the late 1990s. Led by the state-owned economy in competitive industries, it carried out various forms of joint-stock reform, and infiltrated and integrated with various non-state-owned capital. Maturity: the Third Plenary Session of the 18th CPC Central Committee in 2013 proposed to “actively develop the mixed-ownership economy.” The mixed-ownership reform has experienced “positive development” in 2013, “accelerated development” in 2014, “three causes, three necessities and three noes” in 2015, “shrinking employee stock ownership” in 2016, “deep mixed reform” in 2017, “promotion + improvement” in 2018, “full implementation” in 2019, “three-year action” in 2020, and “integration and development of deep transfer operation mechanism” in 2021, China’s mixed-ownership reform has experienced several rounds of deepening and adjustment and is entering a critical period. These are innovative measures taken by the government to promote the reform of mixed ownership from “top-down” of “enterprise management” to “bottom-up” and “two pronged” of “capital management” reform.

Chinese mixed-ownership reform enterprises are characterized by high input, talent density, innovation, growth, income, and risk, along with a flat organizational structure and an organizational culture of cooperation and sharing. The status of upper echelon members (i.e., senior managers who participate in major strategic decision-making) of Chinese mixed-ownership reform enterprises during COVID-19 has affected and continues to affect companies’ life cycles and psychological capital structures. The psychological capital structure and group decision-making behavior of upper echelons of enterprises in China’s mixed-ownership reform are different. In order to improve the group decision-making performance of upper echelons in Chinese mixed-ownership reform enterprises, group decision-making strategies must be formulated according to different popular stages in order to realize the high-quality development of China’s mixed-ownership reform enterprises.

### Prospect Theory

Prospect theory makes an outstanding contribution to artificial judgment and decision-making for uncertain cases by modifying maximum utility theory. Its core concepts—value function and probability weight function—are constantly refined and perfected. Psychological capital is an important concept in positive psychology. It is based on four qualities of positive psychological power: self-efficacy, optimism, hope, and resilience. Currently, research on psychological capital has developed in two directions: individual and team psychological capital. Based on prospect and cumulative prospect theory, the decision performance reference point of the pandemic is taken as the decision benchmark. Group decision strategy according to different pandemic stages has been previously studied and applied to many fields of economics and management based on evolutionary game and system dynamics. Kim and Kim (1997) and Petia et al. (2000) constructed a mixed strategy game model based on system dynamics and conducted effective simulation analysis.

In this context, decision-making of the team psychological capital of upper echelons should conform to the development of the intelligent era. It must first strengthen the upper echelons’ individual psychological capital. The new leap taken of the team psychological capital of upper echelons will manifest in the individual psychological capital development to critical stage.

### Mechanism of the Psychological Capital Structure of Upper Echelons

On the research of team psychological capital structure, Luthans et al. (2005) and Zhong et al. (2013) pointed out based on the research that the team psychological capital structure of Chinese culture is an extension of the individual psychological capital structure, and the team psychological capital structure can play a more significant role in performance than the individual psychological capital structure. Team psychological capital is an element to promote team growth and development, which is formed on the basis of individual psychological capital development. Team psychological capital structure is a positive psychological state, which is produced in the process of team individual cooperation and communication and plays a positive role in the completion of enterprise performance. Through the interaction, integration and exertion of individual psychological capital, team psychological capital forms the psychological quality and characteristics of the team to improve creativity and integration advantages. The upper echelons psychological capital structure of the mixed reform enterprise is the application of the team psychological capital structure in a specific range. It is a psychological state in which the upper echelons psychological capital is generated in the process of upper echelons communication and cooperation and plays a positive role in the achievement of the objectives of the mixed reform enterprise. The high echelon psychological capital structure of mixed reform enterprises is the focus of the influence mechanism of team psychological capital structure under the background of mixed reform enterprises. The mutual influence process of upper echelons decision-making refers to the process of exchanging information and emotional cognition among upper echelons members according to internal rules. Jiao et al. (2021) pointed out that there is an obvious correlation between the psychological capital structure of the upper echelons of the mixed reform enterprise and its decision-making behavior by studying the impact of the four dimensions of the psychological capital structure of the upper echelons of the mixed reform enterprise on decision-making. In the psychological capital structure of upper echelons, upper echelons members with a strong sense of hope can form decisions through performance goal orientation, and organize, lead and control the promotion of the plan. These upper echelons members can dynamically adjust themselves and their emotions and behaviors toward upper echelons. High level echelon members with good toughness can integrate their own experience and resources and promote the development of the organization through dynamic authorization mechanism. Existing research shows that team psychological capital is not the simple summation of individual psychological capital, but comes from the integration and interaction of individual psychological capital. Similarly, the interaction between individual psychological capital and team psychological capital needs to be explored.

**Figure 1:**
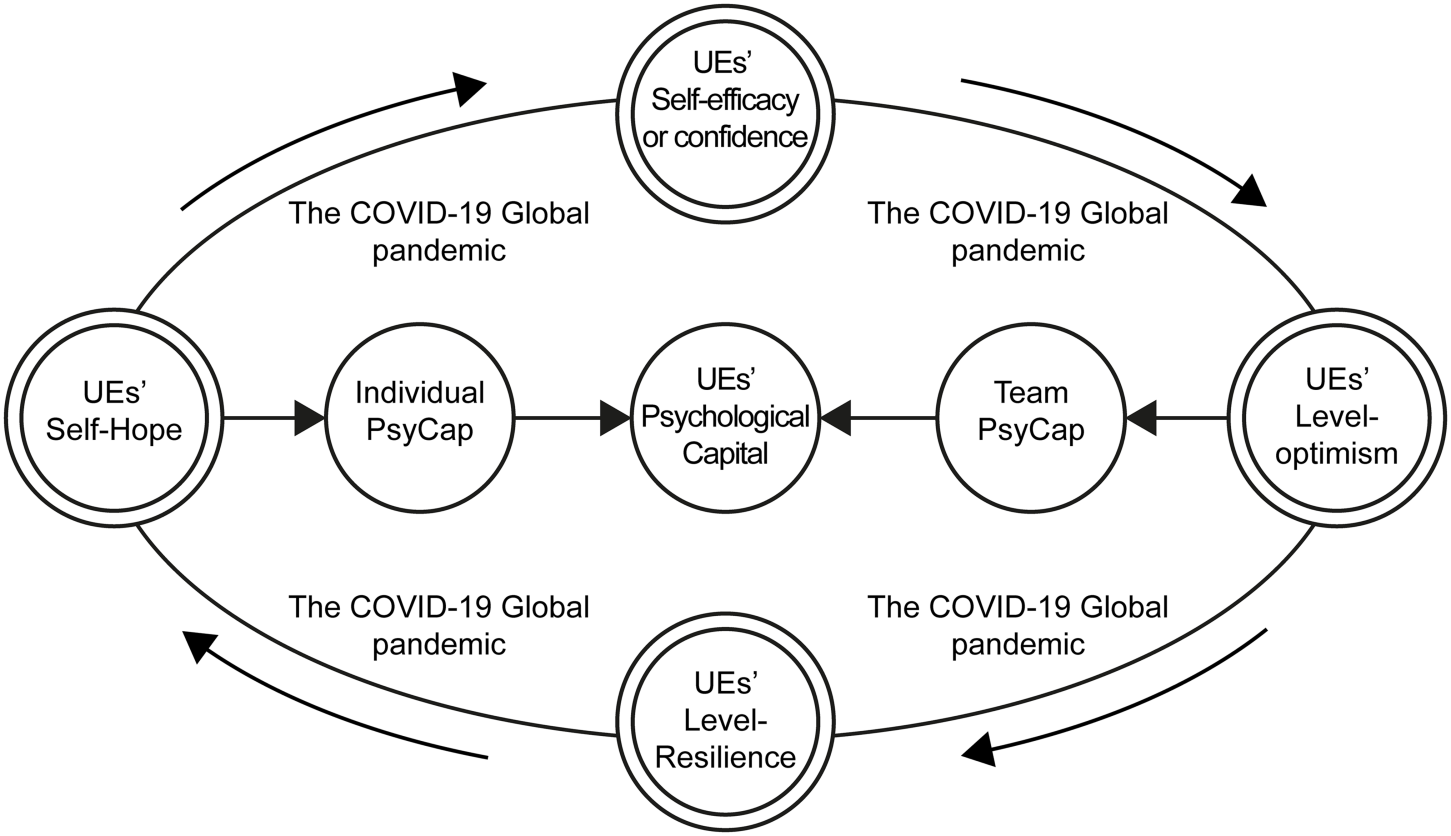
Mechanism of the psychological capital structure of upper echelons.

To sum up, the interaction between “efficacy,” “Hope,” “optimism,” and “resilience” and work performance in the psychological capital structure of the upper echelons of the mixed reform enterprise is a dynamic adjustment process. The psychological capital structure affects the financing development performance of the mixed reform enterprise by affecting the reconstruction of the power structure and decision-making behavior of the upper echelons. Based on the complexity of mixed reform enterprises and the above research, the system dynamics model can be used to study its path and effect on power structure and development performance. Upper echelons should be more inclined toward analyzing the influence of the psychological capital on performance and studying the internal driving mechanism of the various elements to examine its formation. The high-performance work system and its dimensions have a positive impact on psychological capital, which can have a direct or indirect positive impact on employees’ work behavior. Figure 1 shows the psychological capital structure of upper echelons during COVID-19.

## Methods and Model

Sommer et al. (2016) state that during crises, organizational practices are turbulent, and an individual’s interest is often at odds with collective interests. Decision-making during a crisis is difficult. Combined with the characteristics of distributed organizations based on prospect theory, this study divides the decision-making process of Chinese mixed-ownership reform enterprises into individual and group decision-making behavior. According to a general overview of the literature on cognition and decision-making, the psychological and behavioral analysis of decision-makers can be divided into individual decision-making behavior analysis (behavior deviation caused by individual factors and the influence of deviation on the system) and group decision-making behavior analysis (overall behavior deviation owing to the interaction between decision-makers), especially in the context of the pandemic, which is worthy of further study. Zhao et al. (2022) state that Professional self-efficacy and subjective wellbeing have a significant positive impact on job performance; Subjective wellbeing plays a complete mediating role between organizational support and job performance, and subjective wellbeing plays a partial mediating role between professional self-efficacy and job performance; Compared with the sense of organizational support, the positive effect of self-efficacy on job performance is more significant.

Decision-making is the reflection of the changes in the internal and external environment of the organization. It demonstrates the consistency between the organization’s internal and external environment, and the organization’s ability, which is crucial for the organization’s production and development. In the field of real decision-making, to avoid the impact of a single decision-maker’s subjective judgment, choice, and preference on the decision-making results, it is often necessary to integrate the group’s experience and wisdom and adopt joint decision-making to analyze, judge, and make decisions. On the one hand, the form of group decision-making is the core force for organizations to diagnose, deal with, and solve unstructured decision-making problems caused by a complex and changeable environment. On the other hand, it is also a powerful guarantee for the satisfaction of decision-making results, and the formation and successful operation of consensus. Many studies have discussed the key factors affecting the performance of group decision-making, such as conflict, trust, and cohesion. However, there are few studies on the impact of team reflexive behavior on decision-making performance.

The upper echelons individual is a complex, changeable and adaptive agent. A complex adaptive system is composed of complex, changeable and adaptive agents. According to the core theory of complex adaptive system: adaptability creates complexity. Upper echelons individuals and teams also show the main characteristics: (1) Upper echelons individuals are active and flexible, which is the key element of complex adaptive system theory. The characteristic of initiative and flexibility is that the initiative of Upper echelons individuals is the main motivation of adaptive system evolution. The degree of individual initiative of Upper echelons determines the complexity of adaptive system, which is also the starting point of complex adaptive system research, evolution and emergence. (2) Upper echelons individuals, teams and the environment (including individuals) interact dynamically. Facing the drastic changes of mixed-ownership reform, individuals, individuals and environment, groups and environment will have dynamic interaction, which is also the initiative of the evolution and evolution of the complex adaptive system of Upper echelons. The complexity of the system is produced in the process of dynamic interaction between individuals and other individuals and environments. The theoretical research of complex adaptive system focuses on the interaction between individual, team and environment. The stronger this interaction is. The evolution of the system is complex and changeable. (3) Complex adaptive system theory connects macro and micro unified design. It can be simulated through the interaction between individuals, teams and the environment in the Upper echelons. If an individual in the Upper echelons has no initiative, or the initiative is very high, then the individual movement and interaction relationship are balanced by statistical theory. In practice, each Upper echelons individual has very high initiative and adaptability. Under the stimulation, drive and balance of the environment and other individuals, it will reflect differences in the interaction of groups. Therefore, the interaction at the micro and macro levels is a new path and method of complex adaptive system theory. (4) The complex adaptive system model introduces random factors, which makes it have stronger vitality and vitality. The entry of random factors is in line with the reality of daily decision-making of Upper echelons. The complex adaptive system theory points out that random factors will not only affect the state, but also affect the organizational structure and behavior. The distribution of random factors will affect the process of system evolution, but the direction of system evolution remains unchanged. The initiative of Upper echelons is to accept lessons, summarize experience, solidify behavior and form preferences. The common characteristics of Upper echelons and complex adaptive system make the Upper echelons based on complex adaptive system theory and other theories more characteristic and new research space.

### New Trends in Chinese Mixed-Ownership Reform Enterprises’ Decision-Making During the COVID-19 Pandemic

During the COVID-19 pandemic’s impact on the economy and the structure and form of economic organizations has been profound. Mergers and acquisitions are frequent for Chinese mixed-ownership reform enterprises, and they involve branches and partners worldwide, frequent business trips, and members working at home and abroad at distributed times and places. Therefore, there is a need for personnel from different regions, who speak various languages, to communicate and coordinate through emerging technologies. The market selection demands a distributed organizational structure. This popularizes the management of mobile communications and mobile office demand. To streamline the organization, improve work efficiency, and reduce office costs, more Chinese mixed-ownership reform enterprises have begun to let employees work from home. Approximately 30 million people in the United States (16–19% of the working population) have been reported to telecommute from home. With the popularity of personal computers and Internet application technology, the use of home offices is growing rapidly. People rely on a computer to access private networks or the Internet to work with organizations and colleagues.

Owing to the economic impact of COVID-19, more organizations will start telecommuting. Chinese mixed-ownership reform enterprises with upper echelons have a distributed organizational structure with geographical isolation, time difference, language, culture, and other characteristics of high autonomy. They are different from the traditional pyramid structure—a flat hierarchical tube. The whole operation of the organizational structure depends on the behavior of each decision-making individual. A distributed organization does not rely on hierarchical management or on the design of complex processes and systems to control the behavior of Chinese mixed-ownership reform enterprises. Unified goals and tasks drive Chinese mixed-ownership reform enterprises’ upper echelons toward spontaneous cooperation. During pandemic crises, such echelons are distributed, and characterized by decentralization, high authorization, high autonomy, and task-based dynamic organization. In such an organization, everyone is an autonomous member. Based on the characteristics of the highly autonomous and heterogeneous network structure of Chinese mixed-ownership reform enterprises, the upper echelon distributes organization members. Therefore, the organization adopts the bottom-up control model, and the decision-making individual of the upper echelon has independent decision-making powers with the full authorization of the organization members. Behavior has a significant impact on group decision-making, while psychological characteristics play an important role in individual decision-making. Prospect theory assumes that a person is finite and rational, and illustrates the thinking process and psychological activities involved in the decision-making process. It can better depict the decision-making performance of Chinese mixed-ownership reform enterprises’ upper echelon members in the face of uncertainty and complexity. The heterogeneity of a distributed organization network forms the decision-making model of higher echelon cooperation and conflict behavior in Chinese mixed-ownership reform enterprises. The research model based on empirical research lacks dynamism because it ignores the influence of time and environment on decision-making, and the dynamic influence of environment on the integration and conflict behavior of decision-makers. This study conducts an in-depth analysis of the decision-making process, and uses a dynamic simulation method to solve the problems caused by the empirical method.

The members of the upper echelon team have different characteristics of knowledge structure, and the resulting cognitive conflict on the reform of mixed ownership has become an inevitable factor in the team process. Although huge cognitive conflict produces interpersonal friction, which negatively affects team effectiveness, this research believes that cognitive conflict is an important factor to promote the information processing and innovation efficiency of upper echelon team members in a complex and turbulent environment. The cognitive reflexivity process of the upper echelon team is the process of member perspective exchange, information sharing, and cognitive conflict escalation. The effect of the emotional reflexivity of the upper echelon team refers to the situation when the degree of cognitive conflict is very high and interpersonal friction among members intense, but team members still focus on work tasks, and have a high degree of cognitive reflexivity. From the perspective of information processing theory, the perception of upper echelon team members of other people’s behavior is an important factor affecting the manner of information interpretation. Therefore, the higher the degree of emotional reflexivity, the less likely the members are to regard constructive cognitive opinions as provocations, which is conducive to the development of cognitive reflexive behavior related to work tasks. The emotional reflexivity of upper echelon teams can effectively deal with interpersonal conflicts among members. It may also reduce bad emotional behaviors (such as sharp language, provocative behavior, and deliberate destruction), and a good team atmosphere is conducive to improving the level of team cognitive reflexivity. High echelon team emotional reflexivity has a buffer effect on interpersonal friction. The opinion conflict in work cognition can easily lead to interpersonal tension. However, emotional investment and introspection will, in essence, interfere with the further deterioration and transformation of interpersonal relationships. When the emotional reflexivity of the upper echelon team is very low, team members are more likely to regard other opinions as personal attacks. In this case, it is very easy to cause information processing deviation and reject the constructive opinions of other members, resulting in a vicious circle that leads to more serious interpersonal friction, and thus a reduction in the team’s cognitive reflexive behavior. When the high echelon team has a high degree of emotional reflexivity, members are more likely to take different opinions as sincere and heartfelt views, rather than as ulterior motives or deliberate personal attacks. This means that the team’s emotional reflexivity can prevent the escalation of bad emotions and promote a good working atmosphere for the high echelon team, which is conducive to the improvement of cognitive reflexivity.

## Decision-Making in Mixed-Ownership Reform

The 19-year-old theory of COD is difficult to be used in international decision-making, and it is difficult to face more complex problems in the field of international economic management. Complex self-adaptive system can describe the decision-making adaptability of the upper echelons of mixed reform enterprises in complex environment. The basic element of the individual composition of the management decision-making system is man, and the basic characteristic of man is sociality, which is complex and changeable. The root of the complex characteristics of management decision-making system is the decreasing (distortion) effect of information transmission between individuals. Especially with the advent of the network era such as we media, the decreasing effect of information transmission will become more and more obvious in human society. The characteristics and advantages of complex adaptive system will also have more and more advantages in the research of management decision-making system. As a derivative subsystem of economy and society, mixed reform enterprise management decision system has strong characteristics of evolution and emergence. It is not only the “black box” of social science research, but also an important field of contemporary complexity science research. The decision-making behavior of upper echelonss has more complex characteristics, but complex adaptive system has a lot of advantages in describing the internal interaction of decision-making behavior system, but there are few achievements in the field of decision-making research of upper echelonss.

To ensure the scientificity and effectiveness of the model, the following assumptions are made.

*Hypothesis 1*: To achieve the group decision-making goal of mixed-ownership reform, the upper echelon needs a stable internal team structure and reorganization. The reorganization of the upper echelon is divided into two stages: group decision-making behavior conflict and behavior integration.

*Hypothesis 2*: The improvement of decision performance is positively correlated with the evolution of upper echelon behavior conflict and integration. Therefore, upper echelon behavior conflict and integration are used to measure the decision performance of mixed-ownership reform.

*Hypothesis 3*: Affected by internal and external factors, the knowledge and experience of the upper echelon will be positively correlated with the change of mixed-ownership reform time and project experience. After reaching a certain critical value, it will begin to decline with the passage of time and number of projects.

**Figure 2:**
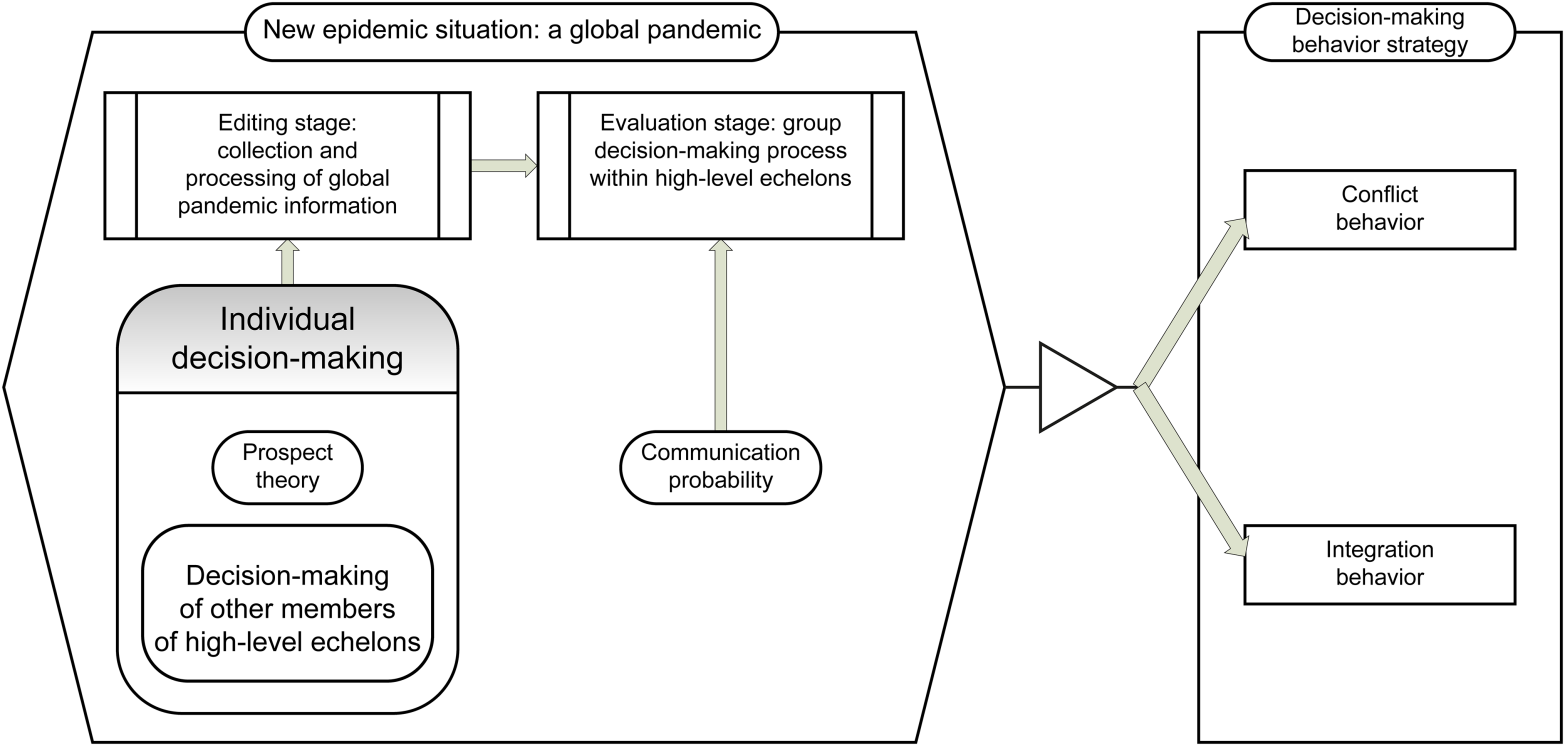
Selection model of the upper echelon conflict and integration behaviors based on prospect theory.

According to Alshammari et al. (2020), social consciousness and relationship management are indispensable skills for leaders in any domain. Restubog et al. (2020) state that emotional intelligence is widely regarded as a central measure that affects job performance. Figure 2 illustrates that, assuming that upper echelon members of Chinese mixed-ownership reform enterprises will communicate *P*, a certain probability, members who adopt a behavior integration strategy will send out information to those who adopt conflict behavior and “persuade” them to adopt behavior integration. When the communication object chooses the upper echelon member of the Chinese mixed-ownership reform enterprise to receive the information, and listens to “persuasion” instead of behavior integration, the behavior changes from the conflict *Con* to *Cin*. However, if the target of communication is to choose the cooperative behavior of upper echelon members, the behavior strategy remains unchanged. To simulate this group’s communication behavior, message transition was added to the *Cin* state. This represents the process of integrating actors to communicate messages (i.e., with *P* as probability) each time the trigger sends an integrated message to a random communication object “persuasion.”

**Figure 3:**
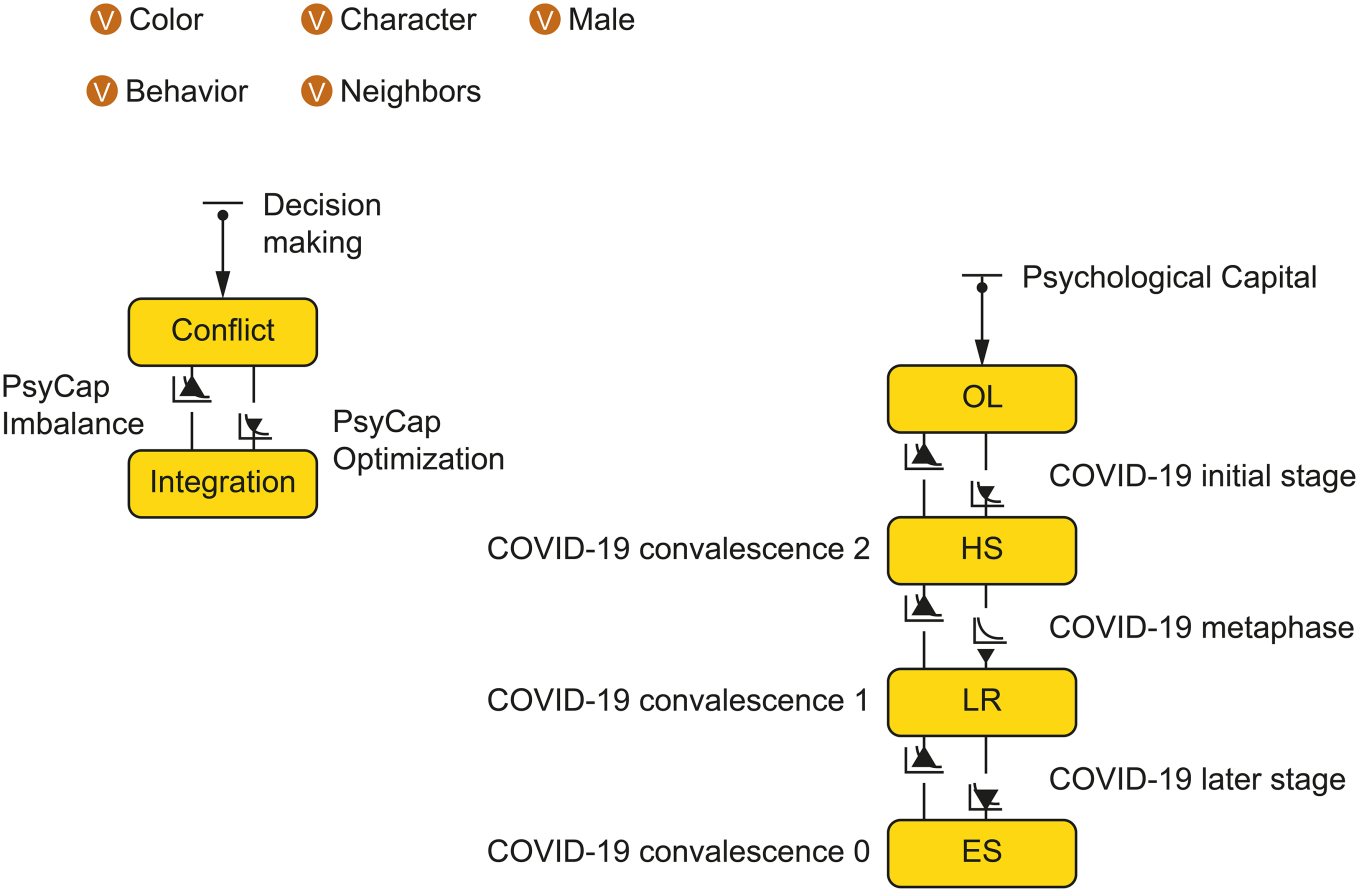
Status chart of the conflict and integration of behaviors affected by COVID-19 Chinese mixed-ownership reform enterprises.

**Figure 4:**
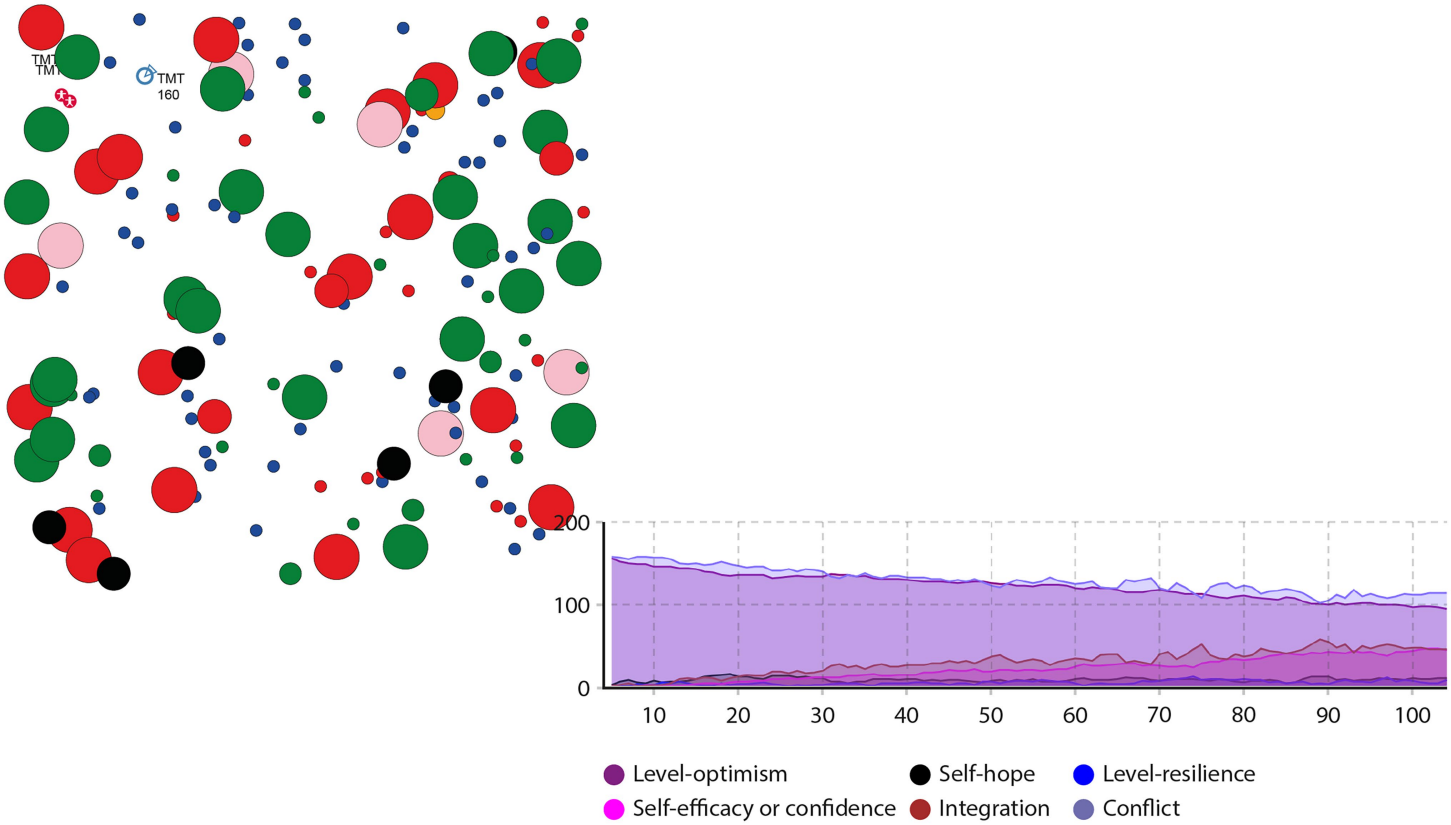
System dynamics analysis model and data.

AnyLogic is a widely used tool for modeling and simulating discrete system dynamics and multi-agent and hybrid systems. It has applications in, logistics, supply chains, economics, business processes, and disease transmission. Suppose the total number of upper echelons of Chinese mixed-ownership reform enterprises in an international group Numberofpeople, is 160, and the income parameters bNumberofpeople is 22, cNumberofpeople is 20, and δNumberofpeople is 8. According to the analysis of senior echelon members’ characteristics in Chinese mixed-ownership reform enterprises during the pandemic, the probability of cooperative behavior and conflict behavior at t = 0 is set at 30 and 70%, respectively. During a pandemic, the initial judgment of Chinese mixed-ownership reform enterprises’ upper echelon members’ psychological capital structure will lose coordination regarding the information on the pandemic and enterprise, and members are likely to demonstrate characteristics of behavior conflict. The decision status of the Chinese mixed-ownership reform enterprise echelons affected by the pandemic is shown in Figures 3, 4.

## Conclusion

The impact of COVID-19 on communication strategies among upper echelons, directly affects the evolution results of the decision-making system. The psychological capital structure in the upper echelons has evolutionary stability strategies in four cases. In the initial stage of the epidemic, the upper echelons power structure and psychological capital structure are in the formation stage. The type of upper echelons power structure has not been determined. The psychological capital structure remains inertial and is in the formation stage, mainly centralized or decentralized power structure. At the beginning of the epidemic, according to the different sources of power and the different ways of authorization, the upper echelons mainly redistributed or competed for power. The power structure and psychological capital structure were in a period of shock, resulting in a restrictive power structure. In the middle of the epidemic, after the redistribution of power and the interaction between power structure and psychological capital structure in the early stage, the upper echelons of mixed reform enterprises will have a stress response and take corresponding measures. At this time, the upper echelons power structure and psychological capital structure will fluctuate repeatedly. During this period, the centralized, decentralized and restrictive power structures will be staggered, but the trend is to reconstruct the restrictive power structure. In the late stage of the epidemic, the upper echelons achieved the expected results through the change of power structure type and the adjustment of psychological capital structure, and the power structure gradually stabilized, showing the characteristics of restrictive power structure.

In some cases, the system evolution presents periodic characteristics. The higher the probability of group communication, the more stable the psychological capital structure, and the greater the fluctuation of behavioral integration. There is a significant correlation between the level of efficacy and resilience of upper echelons psychological capital structure and upper echelons decision-making behavior. The kinetic and potential energies of group decision-making of the upper echelons are not simple summations of the kinetic and potential energies of individual decision-making by the upper echelons, but instead comprise complex conflicts and internal integrations, which need further research. The overall kinetic energy of decision-making by the upper echelons group gradually increases with the advance of decision-making time, while decision-making potential energy gradually decreases with the advance of decision-making time. This indicates that with the increase of behavior integration, behavior conflict within the upper echelons decreases, the small alliance among executives gradually changes into a larger one, and the gap of internal dominant quality h gradually narrows. Upper echelon team conflict management includes implementing strategies to limit the negative effects of conflict and give full play to the positive effects of conflict, as well as improving the effectiveness of the upper echelon team (team learning, organizational behavior, effective communication, and information sharing). Conflict management does not aim to eliminate conflict behavior or avoid conflict behavior, but to employ appropriate management methods to improve team effectiveness. Appropriate conflicts should be restricted to the team and must be managed. The excessive suppression of conflict leads to the reduction of conflict; unmanaged or improper management of conflict will lead to the escalation of conflict or result in an uncontrollable situation. Effective conflict management of upper echelon teams has become an important decisive force to improve team performance and achieve team goals; it is also related to the vitality of the R & D team. It has an important impact on the emotional reflexivity and interactive behavior of upper echelon team members, and has a further impact on the effectiveness of the upper echelon team.

Under the condition of improving the communication probability among upper echelon members, there is a positive correlation between the level of hope and optimism of upper echelon and the power structure of upper echelon and the development performance of mixed reform enterprises. The greater the difference in individual psychological characteristics of team members, the less commonness among members, the more difficult it is for team members to cooperate, and the more common the differences and contradictions between them. The fault of personality psychological characteristics of upper echelon members is divided into two or more sub-teams according to personality psychological characteristics; thus, one group comprises members with high extroversion and a strong sense of responsibility, and the other comprises members with low extroversion and a weak sense of responsibility. For each upper echelon member, the personality and psychological characteristics within the sub-team are more similar than outside the sub-team, so they are more inclined to support the views of members in the sub-team and protect their interests. Once differences develop among team members in the decision-making process, these evolve or devolve easily into sub-team differences, making the conflicts of individual team members into sub-team conflicts. The upper echelons psychological capital structure has a significant regulatory effect on the relationship between the upper echelons power structure and the integration of upper echelons decision-making behavior. For the upper echelons with high levels of resilience, efficacy, optimism and hope of psychological capital structure, there is a significant positive correlation between them; For the upper echelons with low levels of resilience, efficacy, optimism and hope of psychological capital structure, the correlation is not significant. There is a positive correlation between the level of efficacy and resilience of upper echelons psychological capital structure and the power structure of upper echelons and the financing development performance of mixed reform enterprises. Under the condition of improving the communication probability among upper echelons members, there is a positive correlation between the level of hope and optimism of upper echelons and the power structure of upper echelons and the financing development performance of mixed reform enterprises.

Develop the psychological capital structure of upper echelon of mixed reform enterprises, improve the level of financing development decision-making ability and improve decision-making performance. This current research in the Chinese cultural context concludes that Psychological capital structure affects positive cognition, the positive correlation between cognitive conflict and decision-making performance is not strong, and that there is even a negative correlation with high trust level in the decision-making team. Although the role of cognitive conflict in improving decision-making performance is not significant in the Chinese cultural context, and is even considered detrimental to decision-making performance, too few cognitive conflicts will have a more adverse impact on enterprise managers in the Chinese cultural background. This is because too few cognitive conflicts will inhibit the generation of views and cause a herding effect, which is not conducive to improving group decision-making performance. China has a collectivist culture that pays more attention to harmony and maintaining good interpersonal relations. Therefore, people prefer to express different views implicitly. Unless there is a serious confrontation, there are usually no heated debates within Chinese teams due to differences in perspective. Once a relationship conflict develops in the team, it will bring negative effects. Emotional conflict hinders effective communication within the team, and makes the focus of the team deviate from the cognition itself. The team with relationship conflict is disharmonious and inefficient, which will not only affect the quality of team decision-making, but also reduce the satisfaction of the team members with the results of decision-making. Therefore, it is necessary to give full play to the functional role of cognitive conflict and resist the non-functional role of emotional conflict to improve the strategic decision-making performance of upper echelon members. The relationship model exists between cognitive conflict, emotional conflict, and decision performance. Group norms and conflict management behavior are moderating variables. We should pay attention to the moderating effect of team reflexive behavior on the relationship between upper echelon cognitive conflict and strategic decision-making performance.

Upper echelon cognitive conflict, with functional characteristics and cognitive orientation, is an important reason to achieve common goals from different opinions. In strategic decision-making is the key factor leading to high-quality decision-making. Cognitive conflict gives full play to the cognitive heterogeneity of team members, to obtain sufficient information and weigh the advantages and disadvantages of various decision-making schemes. Cognitive conflict is also conducive to the reconstruction of the cognitive basis of high echelon members. Opposing views and ideas provide a more profound, extensive, and accurate cognitive platform for upper echelon members, which can encourage members to think creatively and put forward new opinions. Therefore, cognitive conflict can give full play to the cognitive heterogeneity of upper echelon members and improve the quality of strategic decision-making. In addition, cognitive conflict is also conducive to team members’ understanding of strategic decision-making. Cognitive conflict can help team members better clarify the problems related to the background of strategic decision-making, and provide team members with an overall grasp of the common direction and strength of strategic decision-making. Through the information interaction of different members, cognitive conflict promotes team members’ further understanding of decision-making. Due to high-intensity cognitive conflict, senior echelon team members can better understand their responsibilities, roles and the reasons for power and resource allocation in the implementation of future strategic decisions. Therefore, team members’ understanding of strategic decision-making makes it easier for the decision-making scheme to be correctly implemented. Commitment is required to reduce the possibility of a decision becoming a target because of its negative impact, and increase the possibility of effective implementation of the decision and overcoming resistance. Intense cognitive conflict encourages the upper echelon team members to express their positions, and this opportunity to strongly voice their opinions is closely related to the emotional acceptance of strategic decision-making. High emotional acceptance also means that the team members have a high commitment to strategic decision-making. In addition, cognitive conflict helps team members really understand each other’s views, positions, and interests. Even if the final decision-making scheme cannot meet everyone’s requirements, it can at least consider the interests of as many team members as possible, based on ensuring the overall interests of the enterprise, to improve the average commitment level of the team.

### Discussion on the Shortages and Future Directions

In this study, Including but not limited to computer simulation research using system dynamics, they are all exploratory research on the psychological capital structure of upper echelons of mixed reform enterprises affected by the COVID-19. The follow-up research can further expand the research scope of the power structure and psychological capital structure of upper echelons of the mixed reform enterprise, and analyze the impact of the interaction between the psychological capital structure and the power structure of the upper echelons on development decision-making behavior, stock price and salary of the upper echelons of the mixed reform enterprise. There is also a need to further study the influencing factors of upper echelons decision-making in mixed reform enterprises. If the mixed reform enterprise environment changes too fast and the internal power structure is too centralized, upper echelons will pay too much attention to the loss of power, and the promotion and implementation of high-quality development will be reduced. China’s mixed ownership enterprises are facing a unique institutional and cultural environment, and the formation and operation of upper echelons also have unique characteristics, which are the support of this theoretical research and the limitations of this research. In the next stage, we expect more research on the interaction between the psychological capital structure and the power structure of the high-level echelons in the institutional and cultural environment, so as to provide more systematic theoretical innovation and practical guidance for the effective formation and operation of the high-level echelons and coping with sudden public crises.

## Data Availability Statement

The datasets presented in this study can be found in online repositories. The names of the repository/repositories and accession number(s) can be found in the article/supplementary material.

## Author Contributions

YJ: conceptualization, methodology, validation, formal analysis, investigation, resources, supervision, and project administration. YJ and YG: software. HL: data curation, visualization, and writing—review and editing. YJ, YG, and HL: writing—original draft preparation. YG: funding acquisition. All authors contributed to the article and approved the submitted version.

## Funding

This work was funded by the Humanities and Social Sciences Research planning fund project of the Ministry of Education, “Research on the impact of TMT cognitive evolution based on team life cycle on decision-making performance” (17YJA630020) and Shanghai First-Class Discipline Construction Project “Management Science and Engineering” (S1201YLXK).

## Conflict of Interest

The authors declare that the research was conducted in the absence of any commercial or financial relationships that could be construed as a potential conflict of interest.

## Publisher’s Note

All claims expressed in this article are solely those of the authors and do not necessarily represent those of their affiliated organizations, or those of the publisher, the editors and the reviewers. Any product that may be evaluated in this article, or claim that may be made by its manufacturer, is not guaranteed or endorsed by the publisher.
